# Age-Related Impairments in Immune Cell Efferocytosis and Autophagy Hinder Atherosclerosis Regression

**DOI:** 10.1161/ATVBAHA.124.321662

**Published:** 2025-02-13

**Authors:** Dominique M. Boucher, Sabrina Robichaud, Victoria Lorant, Jonathan S. Leon, Issraa Suliman, Adil Rasheed, Leah I. Susser, Christina Emerton, Michele Geoffrion, Erica De Jong, Dawn M.E. Bowdish, Masanori Aikawa, Elena Aikawa, Sasha A. Singh, Katey J. Rayner, Mireille Ouimet

**Affiliations:** 1 Department of Biochemistry, Microbiology and Immunology, University of Ottawa, ON, Canada (D.M.B., S.R., V.L., I.S., A.R., L.I.S., K.J.R., M.O.).; 2 University of Ottawa Heart Institute, ON, Canada (D.M.B., S.R., V.L., J.S.L., I.S., A.R., L.I.S., C.E., M.G., K.J.R., M.O.).; 3 McMaster Immunology Research Centre, McMaster University, Hamilton, ON, Canada (E.D.J., D.M.E.B.).; 4 Firestone Institute for Respiratory Health, St. Joseph’s Healthcare Hamilton, ON, Canada (E.D.J., D.M.E.B.).; 5 Cardiovascular Division, Department of Medicine, Center for Interdisciplinary Cardiovascular Sciences (M.A., E.A., S.A.S.), Brigham and Women’s Hospital, Harvard Medical School, Boston, MA.; 6 Cardiovascular Division, Department of Medicine, Center for Excellence in Vascular Biology (M.A., E.A., S.A.S.), Brigham and Women’s Hospital, Harvard Medical School, Boston, MA.

**Keywords:** atherosclerosis, cellular senescence, lipoproteins, LDL, macrophages, phagocytosis, plaque, atherosclerotic

## Abstract

**BACKGROUND:**

Aging is a well-established risk factor for the development and progression of atherosclerosis, but the molecular mechanisms underlying this relationship remain poorly defined, and its role in atherosclerosis regression is unknown. To uncover age-related alterations that may impair atherosclerosis regression, we investigated the response of young and old macrophages to atherogenic lipoproteins in vitro and in vivo.

**METHODS:**

Metabolic and proteomic studies were performed in vitro using macrophages differentiated from the bone marrow of young or old mice. To test the role of immune cell aging in atherosclerosis regression, bone marrow from young and old donors was transplanted into irradiated young recipient mice expressing gain-of-function AAV-PCSK9 (adeno-associated virus-proprotein convertase subtilisin/kexin type 9). Following 14 weeks of Western diet feeding, atherosclerosis regression was induced by switching to a standard laboratory diet for 4 weeks.

**RESULTS:**

Compared with young macrophages, old macrophages accumulated more lipid droplets upon lipid loading with the pro-atherogenic lipoprotein aggregated LDL (low-density lipoprotein), accompanied by a failure to proportionally induce autophagy and cholesterol efflux. Proteomic analysis of bone marrow–derived macrophages revealed that pathways related to endocytosis, engulfment, and phagocytosis were downregulated in old lipid-loaded macrophages. Functional studies confirmed a reduction in efferocytic capacity in old macrophages. In recipient mice transplanted with old bone marrow, atherosclerosis regression was impaired, as evidenced by inefficient resolution of circulating inflammatory cell levels, reduced activation of plaque autophagy and apoptotic cell clearance, and persistent plaque CD45^+^ and CD68^+^ content.

**CONCLUSIONS:**

Aging impairs macrophage function through reduced efferocytosis and autophagy activation, limiting atherosclerosis regression. These results highlight the need to better define the mechanisms linking aging to atherosclerosis to develop targeted therapies for the aging population.

**Highlights**

- This study investigates the impact of aging on macrophage metabolic dysfunction and its consequences for atherosclerosis regression, addressing a critical issue given the aging global population.
- Aged macrophages exhibit impaired lipid droplet clearance and reduced autophagy and efferocytosis, key processes essential for resolving atherosclerotic plaques.
- Quantitative proteomics reveal significant downregulation of pathways related to lipid metabolism, endocytosis, and phagocytosis in aged macrophages.
- A bone marrow transplant model demonstrates that young bone marrow enhances plaque remodeling and resolution, whereas old bone marrow fails to provide similar benefits.
- Age-related macrophage dysfunction hinders atherosclerosis regression, underscoring the need for targeted therapies for the aging population.

Atherosclerosis is initiated by the recruitment and infiltration of circulating monocytes in response to pro-atherogenic lipoprotein deposition in the arterial intima.^1^ Within the vessel wall, the uninhibited engulfment of atherogenic lipoproteins by monocyte-derived macrophages results in their storage of excess cholesterol and triglycerides in lipid droplets (LDs).^2,3^ As lipid engulfment exceeds catabolic capacities, macrophages become filled with LDs and become foam cells.^4^ During atherosclerosis progression, lipid-rich plaques within the artery wall gradually develop into advanced vulnerable plaques prone to rupture. A key therapeutic goal is the reduction of plaque size, lipid content, inflammation, and cellular burden, collectively referred to as atherosclerosis regression.^5,6^ Central to this process is the removal of plaque cholesterol through reverse cholesterol transport and the resolution of inflammation.

Autophagy, the lysosomal degradation pathway for cellular components, plays a critical role in maintaining cholesterol homeostasis by mediating the turnover of proteins and organelles, such as LDs.^7,8^ Impaired macrophage autophagy leads to defective LD clearance, reduced cholesterol efflux, impaired efferocytosis, inflammasome activation, and increased macrophage apoptosis^8–11^; collectively, these dysfunctions exacerbate plaque development.^9,10,12,13^ Conversely, activation of autophagy can mitigate atherosclerosis progression by enhancing LD catabolism, cholesterol efflux, and efferocytosis.^14,15^

Macrophage foam cells are a hallmark of atherosclerosis and key integrators of inflammatory and metabolic signals in atherosclerotic plaques.^2^ More recently, vascular smooth muscle cells (VSMCs) within the arterial wall were also found to contribute significantly to foam cell populations.^12,16–19^ Studies have shown that VSMC-derived foam cells comprise 30% to 70% of foam cells in mouse and human atherosclerotic plaques^12,16–19^ and express macrophage markers such as Mac-2 (galectin-3) and CD68 when cholesterol loaded. Distinct arterial foam cell populations can be identified based on CD45 expression, with macrophage foam cells primarily located in lipid-rich CD45^+^ regions and VSMC foam cells in lipid-rich CD45^−^ areas.^12,19^

While hyperlipidemia has historically been considered the primary driver of atherosclerosis, it is now clear that elevated circulating LDL (low-density lipoprotein) alone is insufficient to cause extensive disease.^20^ Atherosclerosis is a multifactorial process involving a plethora of risk factors^21^ including age, which is a strong and clinically relevant determinant of disease development^22^ and may be linked to reduced regression in response to statin therapy.^23^ Aging in the bone marrow increases clonal hematopoiesis of indeterminate potential and VSMC polyclonality, both of which accelerate atherosclerosis.^24–27^ Additionally, aging impairs autophagy, recognized as a hallmark of aging,^28,29^ and contributes to macrophage dysfunction, which is characterized by a primed inflammatory and senescent phenotype, reduced efferocytosis, diminished autophagic flux in response to LPS stimulation, mitochondrial dysfunction, and impaired immune resolution.^30–32^ However, how these age-related alterations in immune cells impact their metabolic responses to atherogenic lipid loading is not well understood, and whether aged immune cells hinder atherosclerosis regression is unknown.

To address this gap, we investigated the metabolic dysfunctions associated with aging in macrophages during atherosclerosis regression. Quantitative proteomic analyses between young and old lipid-loaded macrophages revealed increased associations with lipid accumulation pathways, concomitant with downregulation of endocytosis, engulfment, phagocytosis, and autophagy pathways in old macrophages. Consistent with this, we observed that aged macrophages accumulate more LDs when exposed to pro-atherogenic aggregated LDL (agLDL) and that these cells fail to upregulate autophagy to a similar extent as young macrophages. Moreover, aged macrophages exhibited a notable impairment in efferocytosis capacity relative to their young counterparts. To evaluate the intrinsic effect of aging on immune cells and their role in atherosclerosis, we performed a bone marrow transplant (BMT) experiment in young recipient mice. We observed a greater activation of autophagy, increased apoptotic cell (AC) clearance, and reduction in leukocytes in the plaques of mice reconstituted with young bone marrow undergoing regression as compared with baseline. In contrast, this beneficial plaque remodeling upon atherosclerosis regression was not observed in mice reconstituted with old bone marrow. Our findings indicate that therapeutic approaches aimed at mitigating plaque inflammation, enhancing efferocytosis or promoting reverse cholesterol transport from plaque foam cells to reverse atherosclerosis should be distinctly tailored for aged cells.

## Materials and Methods

### Data Availability

The data supporting the findings of this study are included in the article and Supplemental Material. Any additional data will be made available by the corresponding author upon reasonable request. The mass spectrometry proteomics data have been deposited to the ProteomeXchange Consortium via the PRIDE (Proteomics Identifications Database) partner repository^33^ with the data set identifiers PXD054290 and 10.6019/PXD054290.

### Mice

All procedures were approved by the University of Ottawa Animal Care and Use Committee. Female and male wild-type (C57Bl/6N) mice were acquired from Charles River Laboratories or the National Institute on Aging aged rodent colonies (contractual arrangement with Charles River Laboratories) and kept on a normal laboratory diet (Envigo; 2019) in a temperature and light-controlled environment. Mice were used as young between the age of 10 and 15 weeks, and old between the age of 81 and 104 weeks.

### Cell Culture

To generate bone marrow–derived macrophages (BMDMs), bone marrow was harvested from the long bones of C57Bl/6N mice and cultured for 7 days in DMEM media supplemented with 20% L929 conditioned media (made in-house), 10% fetal bovine serum, and 1% penicillin-streptomycin. Cells were maintained at 37 °C and 5% CO_2_.

### Proteomics

Details on chemicals, protein digestion, liquid chromatography-mass spectrometry analysis, spectral annotation, proteome analyses, and Ingenuity Pathway Analysis can be found in Supplemental Methods.

#### Experimental Design

Young and old BMDMs from individual mice (n=5 old and n=5 young) were treated or not with agLDL at 50 µg/mL for 24 hours, after which the total protein was harvested in RIPA (radioimmunoprecipitation assay) lysis buffer (Pierce) supplemented with Complete Protease Inhibitor (Roche).

#### Spectral Annotation

The peptide spectra corresponding to 20 individual raw files were analyzed using Proteome Discoverer (version 2.4; Thermo Fisher Scientific). The final consensus proteome, therefore, comprised a total of 20 samples corresponding to the young versus old BMDMs, with or without agLDL loading (n=5 replicates per age and treatment). A minimum of at least 2 unique peptides for each protein was required for the protein to be included in the final consensus (4071 proteins).

#### Proteome Analyses

The exported proteome was analyzed in the omics statistical software suite Qlucore (version 3.9; Qlucore; Tables S1 and S2). A 2-group means analysis was done using the statistical software Qlucore Omics Explorer (version 3.7; Qlucore). *P* values were calculated using the Student *t* test^34^ (provided by Qlucore). The 2-group comparisons were (1) the intra-age (young versus old BMDMs) comparisons for the nonloaded group versus the agLDL-loaded group (n=5 each) and the intratreatment (nonloaded versus agLDL loaded) comparisons for old versus young BMDMs groups (n=5 each). Due to a large variability in LD accumulation and individual differences between biological replicates from 2-year-old mice, and our limited access to aged mice, statistical significance was set at *P*<0.1 to capture a broad set of differentially abundant proteins that may be of functional significance. Volcano plots were generated using the statistical cutoff of *P*<0.1.

#### Ingenuity Pathway Analysis

**Figure 1.**
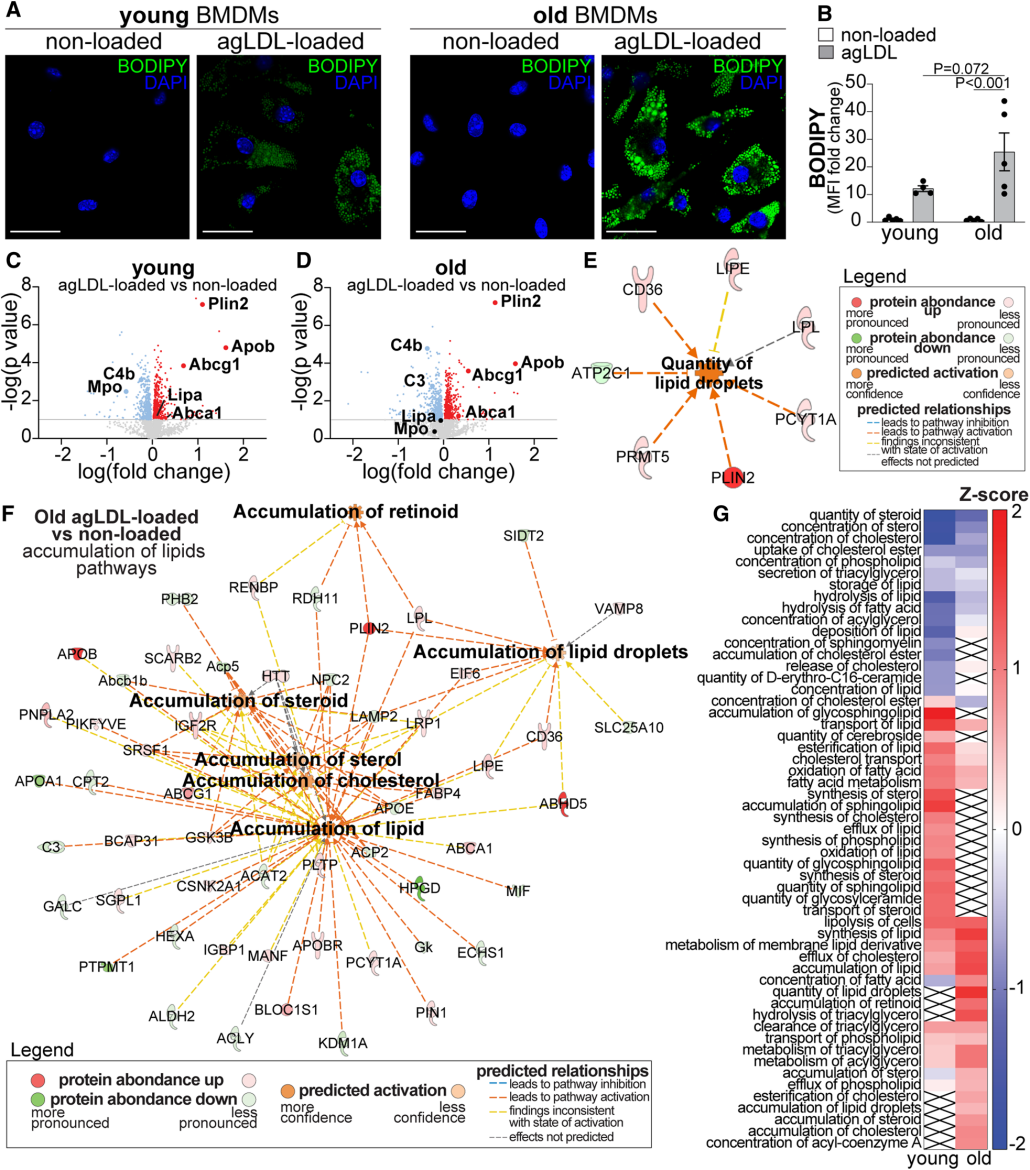
**Old bone marrow–derived macrophages (BMDMs) accumulate more lipid droplets with aggregated low-density lipoprotein (agLDL) loading. A**, Fluorescence microscopy of nonloaded and agLDL-loaded BMDMs from young and old mice. Cells were fixed and stained for BODIPY and DAPI (4′,6-diamidino-2-phenylindole; n=5, BMDMs collected from different mice). Scale bar, 20 µm. **B**, BODIPY quantification from images in **A**. Data are mean±SEM. **C **through **G**, Proteomics analysis of young vs old BMDMs (n=10 per age group), with or without agLDL loading (n=5 agLDL-loaded, n=5 nonloaded per age group). **C **and **D**, Volcano plot of differentially abundant proteins (*P*<0.1) in (**C**) agLDL-loaded vs nonloaded young BMDMs and (**D**) agLDL-loaded vs nonloaded old BMDMs. Proteins more abundant in agLDL-loaded BMDMs are represented in red, and proteins more abundant in nonloaded BMDMs are represented in blue. **E**, Ingenuity pathway analysis (IPA) protein network of the function quantity of lipid droplets in old BMDMs, agLDL-loaded vs nonloaded (*P*<0.1; Z score, 1.671). **F**, IPA pathways of lipid metabolism, relating to the term accumulation (*P*<0.1; |Z score| >0.5). **G**, *Z* score of lipid metabolism–related IPA pathways, comparing agLDL-loaded vs nonloaded young BMDMs (left column) to agLDL-loaded vs nonloaded old BMDMs (right column). Included are pathways with *P*<0.1, |Z score| >0.5 in at least 1 age group. Boxes with an X signify that this pathway was not detected in that age group.

Data were analyzed and generated through the use of QIAGEN Ingenuity Pathway Analysis,^35^ by performing age and treatment comparisons as specified in figure legends between young nonloaded (n=5), young agLDL-loaded (n=5), old nonloaded (n=5), and old agLDL-loaded (n=5) data sets. The Diseases & Functions Analysis identified the biological functions or diseases that were most significant from the data sets. Proteins from the data set that met the *P*<0.1 cutoff were associated with biological functions or diseases in the QIAGEN Knowledge Base and were considered for the analysis. A right-tailed Fisher exact test was used to calculate a *P* value determining the probability that each biological function or disease assigned to that data set is due to chance alone. A *Z* score was calculated to indicate the likelihood of increase or decrease of that disease or function. Lipid metabolism functions were directly interrogated, and comparison between agLDL-loaded versus nonloaded young BMDMs versus agLDL-loaded versus nonloaded old BMDMs was performed to generate the heatmap in Figure 1G. We report those with |*Z* score| >0.5 for at least 1 of the age groups. Furthermore, protein network maps were generated for lipid metabolism pathways under the term of accumulation (Figure 1F; Figure S1B) and other terms (Figures 1E, 5B, and 5C) that met the |*Z* score| >0.5.

### In Vivo Regression Study

Three-month-old male and female mice were lethally irradiated (2×450 cGy, at least 3 hours apart) and transplanted via retro-orbital injection with bone marrow (5×10^6^ cells per mouse) from young (3-month old; n=21 recipients) or old (2-year old; n=26 recipients) male donors. Following a 7-week recovery period, the mice received an intraperitoneal injection of 1×1011 particles of AAV (adeno-associated virus)-gain-of-function PCSK9 (proprotein convertase subtilisin/kexin type 9; University of Pennsylvania) and were started on Western diet (WD; Envigo; TD.88137, 0.2% cholesterol). At 14 weeks of WD feeding, the baseline group was euthanized, and the regression group was switched to a standard laboratory diet (Envigo; 2019) for an additional 4 weeks. All mice were euthanized by exsanguination under isoflurane anesthesia.

### Histological Analyses

All histological analyses were performed and analyzed in accordance with the American Heart Association guidelines.^36^ Briefly, the aortic roots were collected at euthanization and embedded in OCT. Ten-micrometer sections taken 100 µm apart were obtained and stored at −20 °C until use. Hematoxylin and eosin stain (Sigma), oil red O (Sigma), and picrosirius red (Sigma) staining were performed as per the manufacturer’s instructions and as previously^12^ to assess plaque area, necrotic area (defined as acellular areas, as represented in Figure S3C), neutral lipid content, and collagen deposition, respectively. Hematoxylin and eosin and oil red O slides were imaged on the Leica Aperio 8 slide scanner with an HC (high capacity) Plan-Apochromat 20×/0.8 objective, and picrosirius red–stained sections were imaged with an Olympus BX50 polarized light microscope with a UPlanFl 10×/0.30 objective.

### Immunofluorescence Microscopy

Slides were fixed in 4% paraformaldehyde (Thermo Fisher Scientific), then incubated with anti-p-ATG16L1 (phosphorylated autophagy-related 16-like 1; rabbit anti-mouse; Abcam) and anti-CD45 (rat anti-mouse; Novus Biologicals) overnight at 4 °C. Secondary antibody staining, AUTODOT Visualization Dye (Abcepta), and counterstaining with DRAQ5 (deep red anthraquinone 5; BioLegend) were performed the next day. Slides were imaged on the Leica Aperio 8 slide scanner with an HC Plan-Apochromat 20×/0.8 objective. Quantification was performed using the Fiji software, as represented in Figure S7.^37^

### In Vivo Efferocytosis

Slides were stained for CD68 and TUNEL, and counterstained with DAPI (4′,6-diamidino-2-phenylindole; BioLegend), before imaging on Zeiss Axio Scan.Z1 using a Plan-Apochromat 20×/0.8 M27 objective. In situ efferocytosis was quantified as described previously.^38^ Briefly, macrophage-associated ACs (TUNEL^+^ nuclei in contact with a CD68^+^ cell) and free ACs (TUNEL^+^ nuclei not in contact with a CD68^+^ cell) were manually quantified in atherosclerotic lesions from a minimum of 3 sections (100 µm apart) of the aortic root. Efferocytosis is represented as a ratio of free AC:CD68^+^-associated AC.

### Statistical Analyses

**Figure 2.**
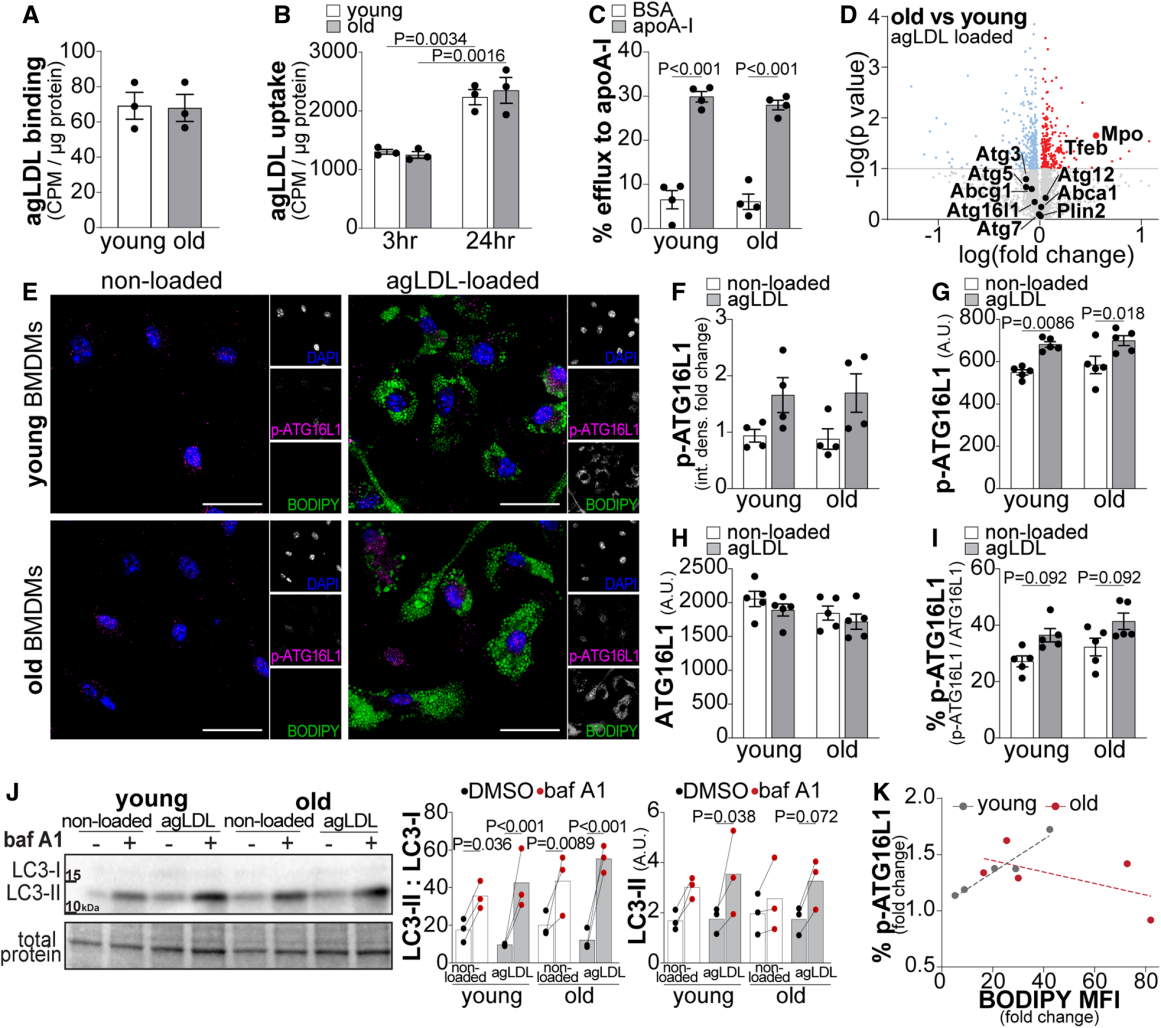
**Aged macrophages show a reduced ratio of autophagy activation relative to lipid loading. A**, Binding of aggregated low-density lipoprotein (agLDL; radiolabeled with ^3^H-cholesterol) by young and old bone marrow–derived macrophages (BMDMs) at 4 °C for 1 hour, as counts per minute (CPM)/µg cell protein (n=3). **B**, Uptake of agLDL (radiolabeled with ^3^H-cholesterol) by young and old BMDMs at 37 °C for 3 and 24 hours, as CPM/µg cell protein (n=3). **C**, ^3^H-cholesterol efflux to apoAI for 24 hours in nonloaded and agLDL-loaded BMDMs (n=4). **D**, Volcano plot of differentially abundant proteins in old vs young agLDL-loaded BMDMs (*P*<0.1; protein more abundant in old in red, and more abundant in young in blue). **E**, Fluorescence microscopy of BODIPY, p-ATG16L1 (phosphorylated autophagy-related 16-like 1), and DAPI (4′,6-diamidino-2-phenylindole) in nonloaded and agLDL-loaded BMDMs from young and old mice (n=4, BMDMs collected from different mice). Scale bar, 20 µm. **F**, Quantification of p-ATG16L1 integrated density from images in **E** (n=4). **G **through **I**, Quantification of p-ATG16L1 (**G**) and total ATG16L1 (autophagy-related 16-like 1; **H**) by AlphaLISA SureFire Ultra assay (n=5) and percent of phosphorylated ATG16L1 (**I**). **J**, Autophagy in nonloaded vs agLDL-loaded BMDMs, ±bafilomycin A1 (baf A1). Representative Western blot of microtubule-associated protein 1A/1B light chain 3 (LC3-I)/LC3-II (n=3). **Bottom**, Quantification of the LC3-II:LC3-I ratio (**left**) and quantification of LC3-II normalized to the adjusted total band volume, in arbitrary units (AU; **right**), from n=3 independent experiments. **K**, Correlation between fold change in BODIPY mean fluorescence intensity (MFI; Figure 1B; BODIPY MFI agLDL-loaded/BODIPY MFI nonloaded) and fold change in percentage of ATG16L1 phosphorylation (Figure 2I; %p-ATG16L1 agLDL-loaded/%p-ATG16L1 nonloaded) in young and old BMDMs (n=5). Simple linear regression in young as gray dotted line, R^2^=0.9357 (slope significantly nonzero, *P*=0.0071), and in old as red dotted line, R^2^=0.3495 (slope significantly nonzero, *P*=0.29). The young and old slopes are significantly different between each other (*P*=0.023). **A **through **K**, Data are mean±SEM.

Data are represented as mean±SEM. The statistical significance was assessed on the GraphPad Prism V10.2.2 software (GraphPad Software, Inc) using 2-way ANOVA with Holm-Sidak multiple comparison test (Figures 1B, 2B, 2C, 2F through 2J, 3B through 3K, 4B through 4D, 5G, and 5H) or unpaired 2-tailed *t* test (Figures 2A and 5D). Outliers were identified and removed using the ROUT (Robust Regression and Outlier Removal) method (Q=1%). For Figure 2K, simple linear regression and statistics were also computed on the GraphPad Prism V10.2.2 software; R^2^, *P* value testing that the slope is significantly different from zero, and 2-tailed *P* value testing of whether the slopes are significantly different are presented.^39^ The number of independent experiments is included in figure legends; when unspecified, data are representative of ≥3 independent experiments conducted with cells harvested from different animals (3–5 animals per age group per independent experiment; Figures 1B, 2B through 2G, 5D, and 5E).

**Figure 3.**
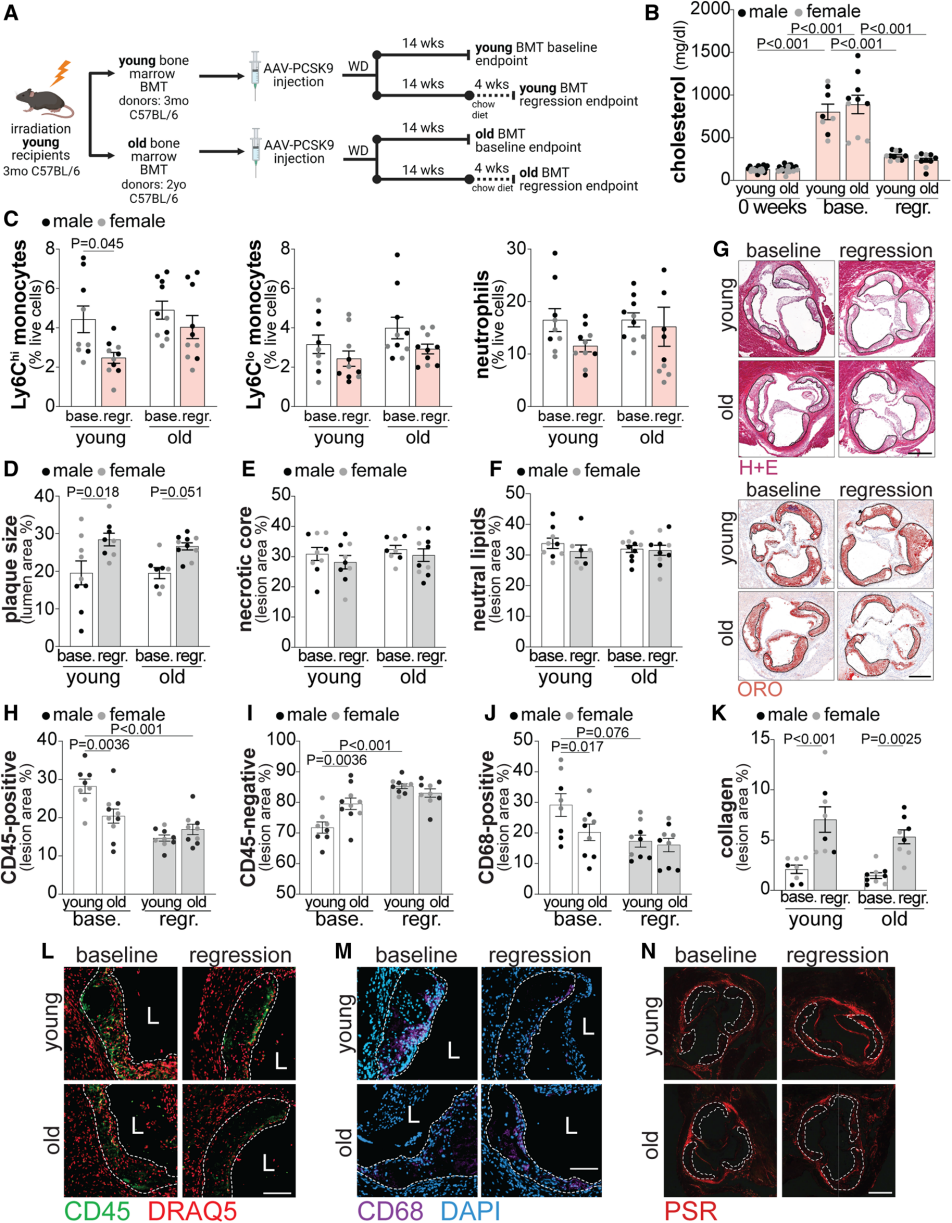
**Impaired measures of atherosclerosis regression in mice with aged bone marrow. A**, Study experimental outline. **B**, Plasma cholesterol levels. **C**, Circulating immune cell subtypes: inflammatory monocytes CD11b^+^ Ly6G^−^ Ly6C^hi^; patrolling monocytes CD11b^+^ Ly6G^−^ Ly6C^lo^; neutrophils CD11b^+^ Ly6C^+^ Ly6G^+^. **D **through **N**, Analyses of atherosclerotic lesions in the aortic root (n=4–6 mice per sex, per group). **D**, Total lesion area. **E**, Necrotic core area. **F**, Neutral lipids. **G**, Representative images of hematoxylin and eosin (H+E) staining (scale bar, 400 µm) and oil red O (ORO) staining (scale bar, 400 µm). **H**, CD45-positive lesion area as a percentage of the cellular lesion area. **I**, CD45-negative lesion area as a percentage of the cellular lesion area. **J**, CD68-positive lesion area as a percentage of the cellular lesion area. **K**, Collagen quantified from picrosirius red (PSR)–stained sections. **L**, CD45 staining (scale bar, 100 µm). **M**, CD68 staining (scale bar, 100 µm). **N**, PSR staining (scale bar, 400 µm). **A **through **N**, Data are mean±SEM.

**Figure 4.**
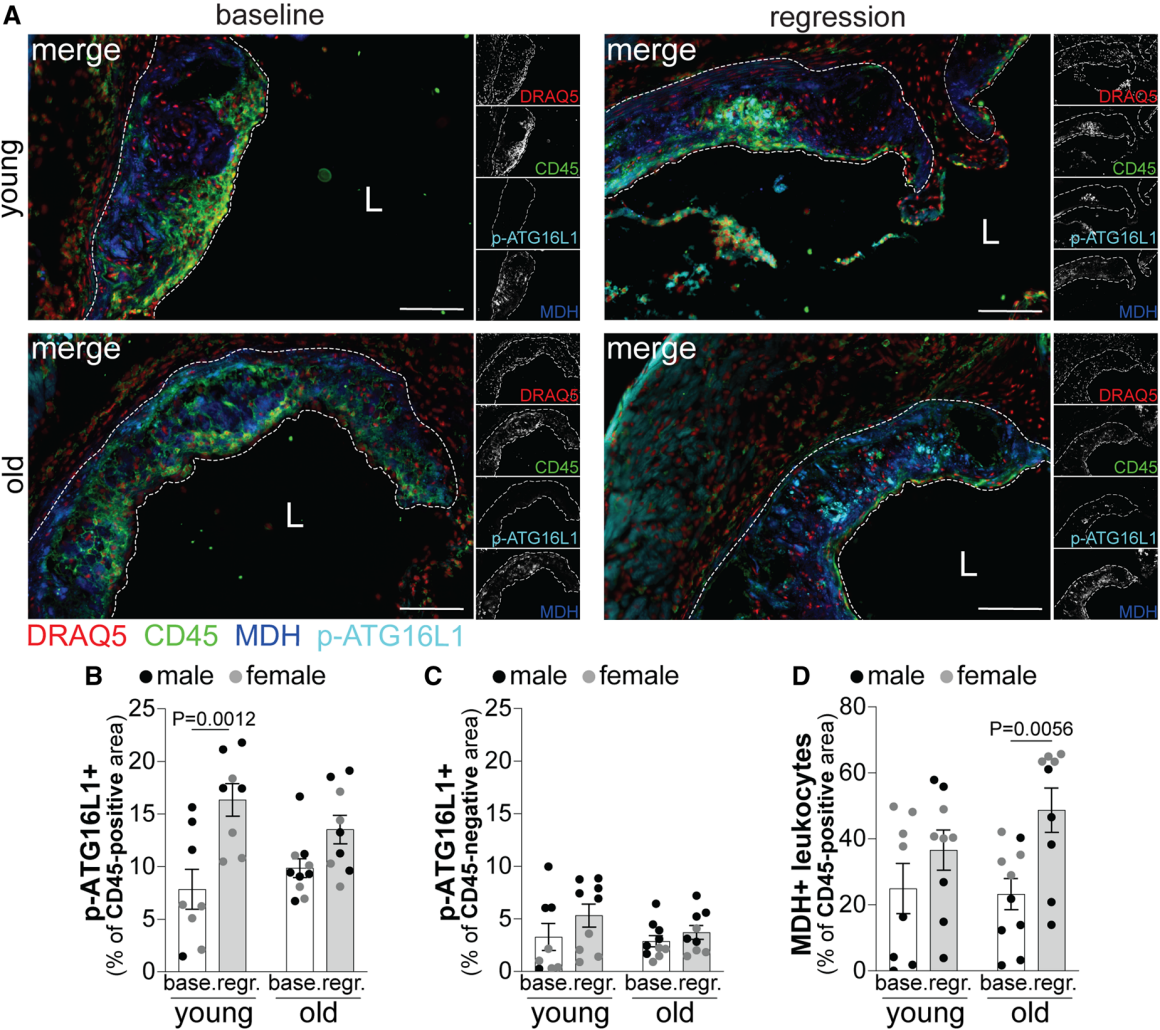
**Impaired autophagy activation in leukocytes from lesions of old regressing mice. A**, Representative images of lesions stained for immunofluorescence microscopy using anti-CD45, anti-p-ATG16L1 (phosphorylated autophagy-related 16-like 1), MDH (monodansylpentane; neutral lipids), and DRAQ5 (deep red anthraquinone 5; nucleus). Scale bar, 50 µm. **B**, Quantification of p-ATG16L1 in CD45-positive area, as percentage of CD45-positive area. **C**, Quantification of p-ATG16L1 in CD45-negative cellular area, as percentage of CD45-negative cellular area. **D**, Percentage of foamy leukocytes, defined as MDH^+^ CD45^+^ cells. Data represented as percentage of total CD45 area. **A **through **D**, Data are mean±SEM.

**Figure 5.**
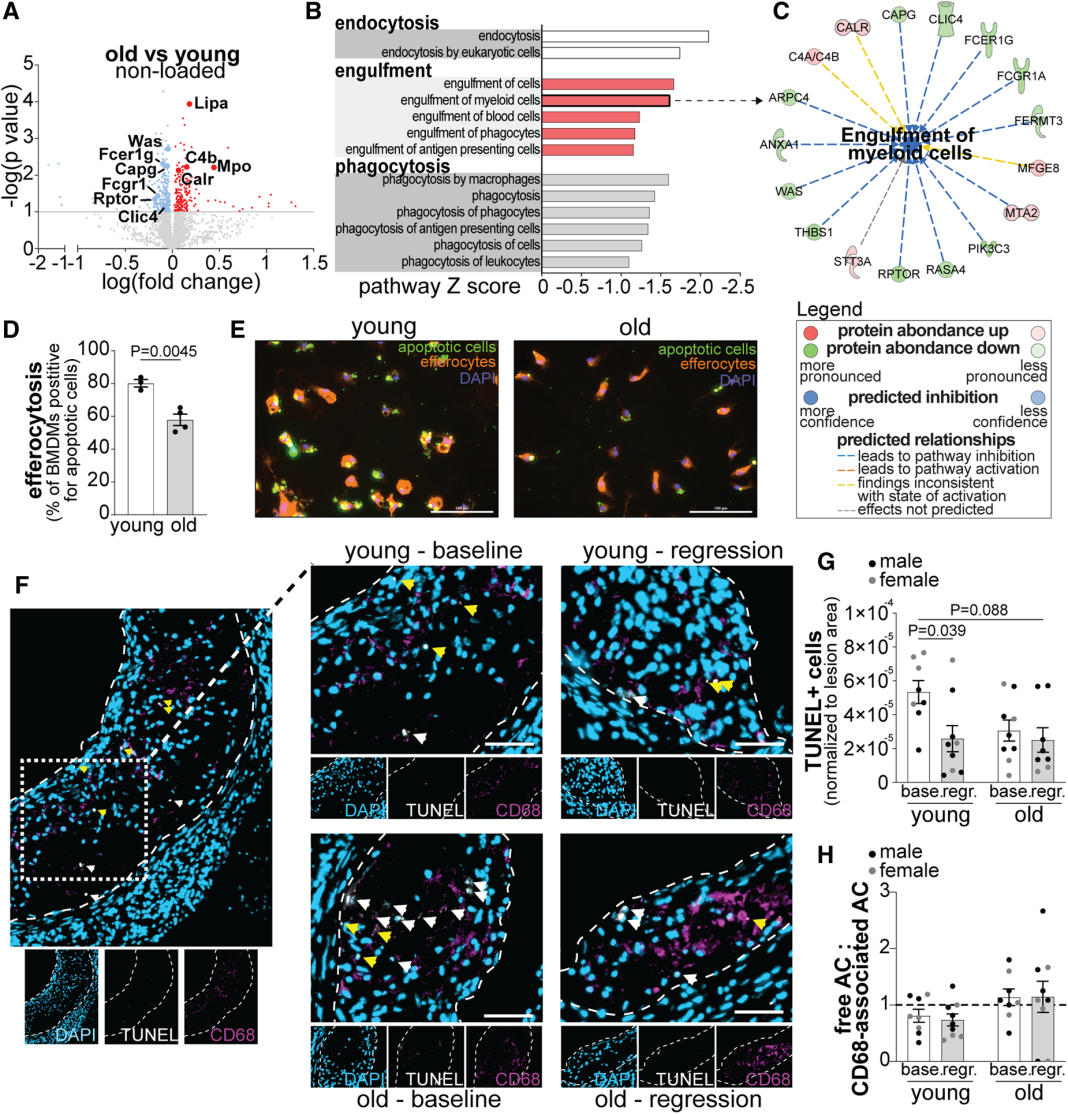
**Efferocytosis is impaired in old macrophages in vitro and in vivo. A**, Volcano plot of differentially abundant proteins (*P*<0.1) in old vs young nonloaded bone marrow–derived macrophages (BMDMs; protein more abundant in old and young in red and blue, respectively). **B**, Comparison of pathways relating to endocytosis, engulfment, and phagocytosis in young and old nonloaded BMDMs, where a negative Z score means lesser expression in old cells. **C**, Protein network relating to the engulfment of myeloid cells in old BMDMs. **D**, In vitro efferocytosis assay, expressed as the percentage of BMDMs that have taken up apoptotic cells (ACs). n=4, BMDMs collected from different mice. **E**, Representative images of in vitro efferocytosis assay. Scale bar, 100 μm. **F**, Representative images of lesions stained for immunofluorescence microscopy using CD68, TUNEL (terminal deoxynucleotidyl transferase dUTP nick-end labeling), and DAPI (4′,6-diamidino-2-phenylindole). Efferocytosis events were defined as macrophage-associated TUNEL-positive ACs (yellow arrows) and distinguished from free AC not associated with a macrophage (white arrows). Scale bar, 50 µm. **G**, TUNEL-positive cells, normalized to lesion area. **H**, Ratio of free AC: CD68^+^-associated AC (efferocytosis events). Values <1 indicate a larger proportion of efferocytosis events. **D **through **H**, Data are mean±SEM.

## Results

### Old Macrophages Accumulate More LDs and Fail to Proportionally Induce Autophagy

To compare the response of young and old macrophages to pro-atherogenic lipids, we incubated macrophages differentiated from the bone marrow of young (3-month old) or old (22-month old) mice with agLDL for 24 hours. Neutral lipids were identified using the fluorescent dye BODIPY, which revealed that old BMDMs accumulate more LDs upon agLDL loading (Figure 1A and 1B). To compare the cellular response to lipid loading of young and old BMDMs, we next performed quantitative proteomics analyses (Figure 1C and 1D). Comparison between agLDL-loaded and nonloaded young BMDMs revealed 507 proteins with increased abundance and 484 proteins with decreased abundance. In old BMDMs, 539 proteins had increased abundance and 486 had decreased abundance. Direct interrogation of pathways relating to lipid metabolism using the Ingenuity Pathway Analysis software found the function quantity of LDs to be significantly changed in old agLDL-loaded BMDMs when compared to their unloaded counterparts, but this was not observed in young BMDMs (Figure 1E). Furthermore, greater associations with lipid metabolism pathways associated with the term accumulation were found in old BMDMs (Figure 1F) than in young BMDMs (Figure S1B) when performing intra-age comparisons between nonloaded and agLDL-loaded conditions. As expected, we observed increased abundance of proteins related to lipid storage (eg, Plin2 [perilipin 2]; *P*=8.46×^10−8^) with lipid loading in both old and young BMDMs (Figure 1C and 1D). In agreement with the observed increase in LD content in old foam cells as compared with young foam cells (Figure 1A and 1B), the functions accumulation of LDs and quantity of LDs were found to be significant in old BMDMs but not in young BMDMs (Figure 1E and 1G). Consistent with this, we observed increased LD numbers and size in old as compared with young macrophage foam cells (Figure S1C through S1E). Moreover, differential regulation was found in many functions related to lipid metabolism (Figure 1G).

This greater accumulation of LDs was not due to differences in the binding (Figure 2A) or uptake (Figure 2B) of agLDL, as these were comparable between young and old BMDMs. We next examined whether the increased accumulation of cytosolic LDs in old macrophage foam cells was due to reduced cholesterol efflux. After LD degradation, free cholesterol is transferred to extracellular cholesterol acceptors, such as lipid-poor APOA1.^8^ In BMDMs loaded with radiolabeled agLDL, we observed no significant difference in cholesterol efflux between young and old macrophages (Figure 2C). Moreover, our proteomic analysis revealed no significant differences in the abundance of key cholesterol transporters, including ABCA1 (ATP-binding cassette transporter A1) and ABCG1 (ATP-binding cassette transporter G1), between old versus young agLDL-loaded BMDMs (Figure 2D).

Upon comparing protein profiles between old versus young agLDL-loaded BMDMs, we identified 249 proteins more abundant in old and 279 proteins more abundant in young agLDL-loaded BMDMs (Figure 2D). Interestingly, we found that TFEB, a master regulator of autophagy, was more abundant in old cells. Modest differences were found in the abundance of various autophagy-related proteins, including ATG3, ATG5, ATG7, ATG12, and ATG16L1 (autophagy-related 16-like 1; Figure 2D). Among others, ATG5 abundance was modestly lower in old cells, coinciding with previous reports of ATG5 reduction in old cells leading to autophagy dysfunction.^40^ Per the importance of LD-specific autophagy, that is, lipophagy, in LD turnover, we further investigated the functional implications of these observations in vitro as a possible source of lipid accumulation. We hypothesized that old macrophages failed to induce autophagy compared with young macrophages, leading to LD accumulation. To test that hypothesis, we quantified the expression of phosphorylated ATG16L1. A key component of autophagosome biogenesis, p-ATG16L1 localizes to growing phagophores and recruits the ATG5-ATG12 conjugating complex to add a phosphatidylethanolamine group to LC3-I (microtubule-associated protein 1A/1B light chain 3) to form LC3-II. This allows for autophagosome maturation with the tagged cargo, such as LDs. We quantified p-ATG16L1 by microscopy (Figure 2E and 2F) and AlphaLISA SureFire Ultra assay (Figure 2G through 2I).^41^ Surprisingly, we observed a similar increase in p-ATG16L1 in young and old BMDMs by fluorescence microscopy (Figure 2E and 2F) upon 24 hours of agLDL loading. In keeping with this, we found no differences in total ATG16L1 or p-ATG16L1 between age groups by AlphaLISA SureFire Ultra assay (Figure 2G and 2H). Equivalent phosphorylation of ATG16L1 was found when expressed as a percentage of total ATG16L1 (Figure 2I). We also quantified the levels of LC3-II and the ratio of LC3-II:LC3-I, as a measure of autophagic flux, in lipid-loaded cells following treatment with the autophagosome-lysosome fusion inhibitor bafilomycin A1. In line with the observed induction of p-ATG16L1, we observed similar levels of autophagy flux in agLDL-loaded cells from young and old mice (Figure 2J; Figure S2).

This was surprising, given that it would be expected the degree of autophagy induction to be greater in the younger macrophages resulting in the observed reduced lipid content relative to old macrophages. Further analysis of the correlation between autophagy induction (pATG16L1) and lipid content (BODIPY) revealed that, as expected, autophagy induction in response to lipid loading was proportional to the relative accumulation of LDs in young macrophages (positive slope, R^2^=0.9357, *P*=0.0071; Figure 2K). However, this was not the case in old macrophages (negative slope, R^2^=0.3495, *P*=0.2938; Figure 2K). In other words, old macrophages failed to induce autophagy to the expected degree in response to increased LDs, relative to their young counterparts. Therefore, although old macrophages can likely induce autophagy to a similar degree as young macrophages, they fail to further activate sufficient autophagic activity during lipid loading, resulting in increased LD accumulation. Collectively, our results indicate that old macrophages accumulate more lipids and that autophagy and cholesterol efflux in old macrophages are not proportional to their LD accumulation. Due to this disproportion, we conclude that old BMDMs likely have dysfunctional lipid loading–induced autophagy activation.

### Mice With Old Immune Cells Exhibit Impaired Atherosclerosis Regression, Inflammation Resolution, and Autophagy Activation as Compared With Those With Young Immune Cells

We next investigated the functional consequences of increased LD accumulation and blunted lipid loading–induced autophagy activation from aged immune cells during atherosclerosis regression. Male and female C57BL/6N mice were lethally irradiated, and their bone marrow was reconstituted using male donors of either young (3-month old; Y-BMT) or old age (2-year old; O-BMT; Figure 3A). Hypercholesterolemia was then induced with gain-of-function AAV-PCSK9 and WD feeding, as previously described.^42^ In this model, reversal of hyperlipidemia by a switch to standard laboratory diet feeding was shown to reduce monocytosis and to induce measures of plaque regression, as defined by reduced plaque size, reduced content of plaque macrophages (CD68^+^), and increased collagen content.^42,43^ Body weight and plasma cholesterol were monitored during the study (Figure 3B; Figure S3A and S3B). Decreased plasma cholesterol to near baseline levels following the switch to standard laboratory diet feeding was confirmed at 18 weeks (Figure 3B). Importantly, we did not observe changes in circulating cholesterol levels between young and old donor bone marrow at any of the time points. Analysis of the immune cell populations in the peripheral blood was performed after 14 weeks of WD (baseline) and following the 4-week regression period. We observed a significant reduction in the proportion of circulating inflammatory monocytes following the regression period in the Y-BMT mice but not in the O-BMT mice, while patrolling monocytes and neutrophils trended toward reduction in both age groups (Figure 3C). Collectively, a significant shift away from an atherogenic circulatory immunophenotype was observed in the Y-BMT group, while inflammatory monocytes remained elevated in the O-BMT group.

Analysis of atherosclerotic plaques in the aortic root showed no differences between age groups in the plaque size, necrotic core area, and neutral lipid deposition, before or after the regression period (Figure 3D through 3G). However, we found that the systemic decrease in pro-atherogenic monocytes correlated with the decrease in leukocytes observed in aortic root plaques after the regression period. While the Y-BMT plaques were basally richer in CD45^+^ leukocytes and CD68^+^ content, a significant decrease in the CD45^+^ and CD68^+^ plaque composition was observed in the regressing Y-BMT group as compared with its baseline but not in the O-BMT group (Figure 3H through 3J, 3L, and 3M). The O-BMT group showed a larger CD45^−^ area at baseline, an indication that nonimmune cells (CD45^−^) made up a larger proportion of the plaque receiving old bone marrow (Figure 3I). While the collagen content of plaques was significantly increased upon regression as expected, a slightly more appreciable trend is appreciable in the young cohort as compared with the old, an effect driven by female mice (Figure 3K and 3N; Figure S5F).

In accordance with our observations that autophagy is not equivalently induced in young and old macrophages upon pro-atherogenic lipid loading (Figure 2), immunofluorescence analyses of p-ATG16L1 in aortic root plaques revealed a greater autophagy induction during atherosclerosis regression in CD45^+^ leukocytes from the Y-BMT cohort (fold change, 2.04) as compared with the O-BMT cohort (fold change, 1.37; Figure 4A and 4B). This was exclusive to the CD45^+^ plaque area, as minimal modulation of the p-ATG16L1 signal in CD45^−^ plaque area was observed between baseline and regression in both age groups (Figure 4C). Interestingly, we found a larger increase in MDH (monodansylpentane) staining in old as compared with young leukocytes following the regression period (Figure 4D), an effect again driven by female mice (Figure S6A). Together, our study reveals a reduction in atherosclerosis burden—as evidenced by reduced leukocyte plaque content and increased autophagy activation in regressing plaques as compared with baseline—in recipients of young, but not old, bone marrow.

### Aging Impairs Efferocytosis

Given the known roles of autophagy in driving efferocytosis,^11,44^ we next tested whether inadequate autophagy induction in old macrophages may impair their efferocytosis capacity. From our proteomic analysis independently comparing nonloaded old and young BMDMs, 3925 proteins were identified, of which 186 were more abundant in old and 254 were more abundant in young BMDMs (Figure 5A). Pathways relating to endocytosis, engulfment, and phagocytosis were found to be downregulated in old nonloaded BMDMs as compared with young ones (Figure 5B and 5C). To quantify the impact of aging on efferocytosis in mouse macrophages, we fed an equivalent number of young and old apoptotic BMDMs to young and old BMDM efferocytes, respectively. We found that the capacity of BMDMs to internalize ACs was decreased in old BMDMs as compared with young BMDMs (Figure 5D and 5E). TUNEL labeling of ACs within atherosclerotic plaques showed fewer ACs in Y-BMT mice undergoing atherosclerosis regression as compared with their baseline counterparts, while reduced plaque apoptosis was not observed in O-BMT regressing mice (Figure 5F and 5G). At baseline, however, more TUNEL^+^ cells were observed in the Y-BMT group as compared with the O-BMT group (Figure 5G). Direct quantification of plaque efferocytosis in vivo as described previously^38^ showed a trend toward fewer ACs relative to CD68-associated ACs in plaques of Y-BMT mice undergoing regression (Figure 5H), which was not observed in O-BMT regressing mice. Collectively, our findings suggest that impaired autophagy in old macrophages reduces efferocytosis, potentially contributing to decreased AC clearance in aged atherosclerotic plaques.

## Discussion

Aging is unequivocally associated with a plethora of immune and metabolic changes that remain incompletely understood. Macrophages are not exempt from undergoing such age-related alterations, as reviewed previously,^45,46^ but how the aging environment and intrinsic changes interact to bring on such changes remains ill-defined. Old macrophages acquire an assortment of dysfunctions relative to their younger counterparts that contribute to atherosclerosis, including a proinflammatory predisposition, dysregulated cholesterol homeostasis, decreased autophagic capacity, and dysfunctional mitochondrial metabolism.^30,47^ Here, we find that aged macrophages accumulate more lipids as compared with young macrophages, in accordance with previous reports of senescence-related lipid buildup in old monocytes and macrophages.^31,47–50^ Additionally, we observed that old macrophages fail to upregulate autophagy and cholesterol efflux proportionally to the extent of LD biogenesis following agLDL loading. With the implications of these functions in maintaining efferocytosis, it is unsurprising that we identified an impairment in efferocytosis in old BMDMs in vitro, in line with previous similar reports of defective efferocytosis by aged macrophages.^51,52^ Importantly, we found that these observations translate in vivo in our model, as a larger proportion of free ACs to the CD68-associated ACs were found in our O-BMT group.

With previous reports describing how aged mice develop greater atherosclerosis burden than young mice,^32,53,54^ we herein sought to investigate the contribution of the macrophage population to this process, focusing on how the autophagic response and predisposition of aged macrophages to accumulate LDs might control the capacity of plaques to undergo atherosclerosis regression. To specifically dissect the role of aged immune cells in atherosclerosis without the influence of the aged systemic environment, aged vasculature, and aged plaque VSMC foam cells, we performed a BMT experiment in young recipient mice. Reduced monocytosis and plaque macrophage numbers are 2 features of atherosclerosis regression in the gain-of-function AAV-PCSK9 diet switch model.^42^ In accordance with this, we observed reduced circulating inflammatory monocyte levels in the Y-BMT mice after 4 weeks of regression. However, we found that the levels of circulating inflammatory monocytes failed to decrease in the O-BMT mice after 4 weeks of regression, an effect driven by male mice. Concurrently, we observed a significantly greater reduction in plaque leukocyte content following atherosclerosis regression in the Y-BMT cohort than in the O-BMT cohort, though Y-BMT plaques had a greater proportion of leukocytes than O-BMT plaques at baseline. A larger CD45^−^ population in the O-BMT plaques would infer a larger population of VSMCs at baseline (eg, after 14 weeks of diet) in comparison to the Y-BMT. This is in line with a recent report where aged BMT resulted in lesions with a larger, polyclonal VSMC population.^24^ The larger CD45^+^ population present in Y-BMT plaques may indicate that in our model, the leukocytes derived from young bone marrow infiltrated the plaques more readily and underwent more replicative events in situ. On the other hand, the striking reduction in CD45^+^ leukocytes in the Y-BMT cohort following regression might be partially attributable to cell death, as supported by the larger number of TUNEL^+^ cells at baseline, with significant reduction after regression.

Cell death in the Y-BMT group is likely productive in reducing plaque cellular content due to effective efferocytosis. Furthermore, the marked decrease in CD45^+^ and CD68^+^ cell numbers in regressing Y-BMT plaques relative to the reduction in TUNEL^+^ cells that is similar in magnitude supports this notion. However, leukocytes can also egress from established atherosclerotic plaques and return to the circulation during the regression period.^55–59^ The regulation of this process and the extent of its contribution to atherosclerosis regression relative to that of reduction of monocyte recruitment to plaques remains unclear.^56,57,59^ The LXR (liver X receptor) promotes egress of monocyte-derived cells from atherosclerosis plaques through a mechanism involving CCR7 (C-C motif chemokine receptor 7) upregulation.^55^ With a recent report describing a reciprocal activation of autophagy and LXR in a feed-forward loop,^59^ it is possible that higher autophagy activation in Y-BMT plaque leukocytes promoted egress through LXR activation.^60^ Whether young macrophages have a greater capacity to egress than their older counterparts and whether LXR agonists can restore the ability of aged macrophages to exit the plaque remains to be tested.

Combined male and female data show a comparable increase in collagen deposition in both Y-BMT and O-BMT cohorts during regression, suggesting that regression-associated VSMC plaque remodeling occurred to a similar extent. Because VSMCs are the primary source of collagen within the fibrous cap^61^ and VSMCs of all recipient mice were age matched, this observation is unsurprising. However, separate analysis of female cohorts reveals a larger increase in collagen in Y-BMT regressing plaques (Figure S5F). Y-BMT females also had a smaller increase in plaque size after standard laboratory diet switch (Figure S5A), and their plaque leukocytes did not further accumulate lipids during the regression period (Figure S6A). In contrast, the Y-BMT male mice underwent a larger decrease in neutrophils, Ly6C^hi^ monocytes, and Ly6C^lo^ monocytes than female mice (Figure S4B through S4D), suggesting more efficient resolution of the circulating pro-atherogenic phenotype. We thus identify sex differences in the response to hypercholesterolemia reversal between our male and female cohorts, similar to our previous report that autophagy activation via trehalose induces atherosclerosis regression in female but not male mice.^62^

Despite observing parameters suggestive of productive remodeling of atherosclerosis, such as decreased immune cells, increased collagen deposition, and activation of autophagy, we did not achieve true halting or shrinking of plaque area in our model. In fact, plaques continued to enlarge after switching to a standard laboratory diet, irrespective of the age of the bone marrow. The relatively short duration of the regression period, coupled with the diet switch as the sole initiator of the regression process, might partially account for these findings. Alternatively, the cumulative influence of the aged vasculature and the interplay of immune cells with aged VSMCs may have a greater impact on the capacity for plaque regression than the aging of immune cells alone. Vascular aging, associated with increased IL-6 production by VSMCs, has been shown to promote atherogenesis in aged AAV-PCSK9–injected mice fed a WD for 10 weeks, exacerbating necrosis in atherosclerosis plaques of the aortic root.^32^ Additionally, inducing VSMC-specific senescence alone can increase atherosclerotic plaque size and necrotic area.^63^ In our model, which involved transplanting bone marrow from young or old donors into young recipient mice, we did not find differences in plaque size or necrosis between age groups. This supports the idea that the aged vasculature, rather than solely the aging of immune cells, is an important contributor to exacerbated atherogenesis and impaired plaque regression. Future studies are needed to disentangle these complex interactions and identify targeted approaches to address both vascular and immune aging in the context of atherosclerosis.

Immune system reconstitution with aged bone marrow resulted in a blunted autophagy induction in plaque leukocytes during regression. Dysfunctional autophagy promotes atherosclerosis development,^8–10,12,14,15^ while the restoration of autophagy flux and myeloid cell bioenergetics in aged mice can ameliorate various age-associated diseases.^40,64^ Restoring autophagy flux in aged BMDMs notably protected against acute liver injury in mice by promoting polarization to a pro-resolving macrophage phenotype,^40^ which coincidentally is also associated with atherosclerosis regression.^5^ Understanding how autophagy becomes defective in aging and testing how restoring autophagy in plaque leukocytes of O-BMT mice may ameliorate atherosclerosis regression represents an important next step in research. So far, age-related autophagy failure in mouse macrophages has been linked to the reduced expression of LC3B and Atg5 genes due to the hypermethylation of their promoter regions via DNA methyltransferases and is amenable to treatment with (2)-epigallocatechin-3-gallate.^65^ In the context of atherosclerosis, previous work showed that the expression of microRNA-33, a repressor of ABCA1 expression, cholesterol efflux, and autophagy,^14,66,67^ is elevated in atherosclerotic plaques^14^ and aged macrophages in hypercholesterolemic WT mice.^48^ Conversely, microRNA-33 antagonism promotes efferocytosis, lysosomal biogenesis, and degradation of apoptotic material, restoring defective autophagy in atherosclerotic plaques.^14^ MicroRNA-33 inhibition, therefore, represents an attractive therapeutic strategy to boost autophagy in aged patients, along with trehalose,^15,68^ spermidine,^32,69^ and metformin^70^ among others that work to promote autophagy flux in vivo.^71^

Little is known about atherosclerosis regression in the aged environment; yet, reversing atherosclerosis represents a significant unmet clinical need as more and more elderly patients live with cardiovascular disease. Current therapeutic strategies targeting atherosclerosis regression focus on the reduction of cholesterol deposition and inflammation^72^; yet some evidence suggests this may not be as efficient in older individuals.^23^ Here, we found that aged macrophages had dysfunctional autophagy and dysregulated cholesterol homeostasis, which positively correlated with elevated lipid accumulation in these cells as compared with young macrophages both in vitro and in vivo. Mice receiving aged bone marrow exhibited impaired capacity for atherosclerosis regression as compared with those receiving young bone marrow. Collectively, our work highlights age-dependent differences in immune cell metabolism, cholesterol handling, and autophagic flux, providing the framework for better understanding of how aging might impair the reversal of cardiovascular disease in older individuals.

## ARTICLE INFORMATION

### Acknowledgments

The authors thank the University of Ottawa Heart Institute Animal Care and Veterinary Services for technical assistance for the mouse atherosclerosis studies. They thank Dr Dawn Bowdish and Erica De Jong (McMaster University) for providing the bone marrow used in the in vivo bone marrow transplant study. They thank Dr Warren Lee and Karen Fung (St. Michael’s Hospital) for providing the recombinant human APOA1 plasmid and for sharing their recombinant APOA1 purification protocol. They thank the University of Ottawa Faculty of Medicine Cell Biology and Image Acquisition Core (RRID [Research Resource Identifier]: SCR_021845), funded by the University of Ottawa, Ottawa, Natural Sciences and Engineering Research Council of Canada, and the Canada Foundation for Innovation, and the Louise Pelletier Histology Core Facility (RRID: SCR_021737) for their assistance with aortic sinus immunofluorescence microscopy. They thank the University of Ottawa Heart Institute High Resolution Cell Imaging Core Facility for their help with aortic sinus histology.

### Sources of Funding

This work was supported by the Canadian Institutes for Health Research (PJT 175214 to M. Ouimet and K.J. Rayner and Canada Research Chair to M. Ouimet), the Canada Graduate Scholarship-Master’s Program (D.M. Boucher), the Queen Elizabeth II Scholarship (L.I. Susser), the University of Ottawa Cardiac Endowment Fund (D.M. Boucher, A. Rasheed, and L.I. Susser), the Vanier Canada Graduate Scholarship (D.M. Boucher and S. Robichaud), and the National Institutes of Health grants (R01 HL136431, R01 HL147095, and R01 HL141917 to E. Aikawa).

### Disclosures

None.

### Supplemental Material

Supplemental Methods

Tables S1 and S2

Figures S1–S7

Major Resources Table

Uncropped Western Blots
